# Real-World Data and Causal Machine Learning to Enhance Drug Development

**DOI:** 10.1007/s43441-025-00849-0

**Published:** 2025-08-02

**Authors:** Chris Anagnostopoulos, Mihaela Van Der Schaar, Jean-Paul Collet, Ramon Hernandez Vecino

**Affiliations:** 1 QuantumBlack, The Post Building, 100 Museum St, Holborn, London, UK; 2 Statistics Section, Imperial College London, London, UK; 3 Department of Applied Mathematics and Theoretical Physics, University of Cambridge, Cambridge, UK; 4 The Alan Turing Institute, London, UK; 5 Evidinno Outcomes Research Inc., Vancouver, Canada; 6 University of British Columbia (UBC), Vancouver, Canada; 7 Department of Research and Development, Sanofi, Gentilly, France

## Abstract

The current paradigm of clinical drug development, which predominantly relies on traditional randomized controlled trials (RCTs), is increasingly challenged by inefficiencies, escalating costs, and limited generalizability. Concurrent advancements in biomedical research, big data analytics, and artificial intelligence have allowed for the integration of real-world data (RWD) with causal machine learning (CML) techniques to address some of these limitations. This manuscript reviews the emerging role of RWD/CML in enhancing clinical research and drug development programs. By leveraging diverse data sources — including electronic health records, wearable devices, and patient registries — CML methods facilitate robust drug effect estimation, enable precise identification of responders, and support adaptive trial designs. Approaches such as advanced propensity score modelling, outcome regression, and Bayesian inference can help mitigate confounding and biases inherent in observational data, thereby strengthening the validity of causal inference. However, these innovative methodologies also face significant challenges related to data quality, computational scalability, and the absence of standardized validation protocols. Furthermore, ethical and regulatory concerns regarding model transparency and validity, data privacy, and possible algorithmic biases stress the importance of multidisciplinary collaboration and rigorous oversight. Our analysis underscores that while RWD/CML integration can enhance clinical development programs by generating more comprehensive evidence and accelerating drug innovation, its successful adoption depends on overcoming technical, operational, and scientific hurdles while maintaining a transparent approach with regulatory agencies.

## Introduction

Drug development is complex, time-intensive, and costly, requiring rigorous testing to ensure efficacy and safety. The process begins with identifying promising molecules, followed by extensive clinical trials and strict regulatory oversight from agencies like the FDA and EMA. On average, it takes 10–13 years to bring a drug to market, with only 1 in 10,000 candidates gaining approval [1–3]. Development costs range from $1–2.3 billion [3–5], contributing to a decline in return-on-investment from 10.1% in 2010 to 1.8% in 2019 [5]. These challenges have driven efforts to streamline molecule selection and improve trial efficiency.

Randomized clinical trials (RCTs) remain the gold standard for evaluating safety and efficacy but have limitations. Phase 3 trials, despite larger samples, still struggle with diversity [6], underrepresentation of high-risk patients [7], and potential overestimation of effectiveness due to controlled conditions [8]. Small sample sizes hinder subgroup analyses and rare adverse event detection. Randomization does not guarantee perfect covariate balance [9, 10], and differential loss to follow-up may introduce biases [11]. Additionally, reliance on surrogate endpoints, such as progression free survival over overall survival, is common—70% of recent FDA oncology approvals used non-overall survival endpoints—raising concerns about real-world relevance [12]. Similar challenges apply to assessing long-term side effects and competing risks in patients with comorbidities [13]. 

To address the limitations of conventional trials, researchers have explored strategies such as decentralized clinical trials, master protocols (basket, umbrella, and platform trials), adaptive designs, patient-centred trials, and the integration of real-world data (RWD) to complement trials for drug development. For instance, RWD captures patient journeys, disease progression, and treatment responses, offering valuable insights into drug efficacy and safety beyond controlled trial settings [14]. It encompasses diverse sources, including electronic health records (EHRs), insurance claims, and structured patient registries—where data follow predefined protocols but without randomized interventions [15–17]. Furthermore, by generating real-world evidence (RWE), RWD plays a critical role in optimizing trial design, improving recruitment efficiency, optimal timepoints for the outcomes and identifying treatment-effect modifiers to support personalized medicine [18]. 

Despite its advantages, RWD presents challenges due to its observational nature and lack of randomization, making it prone to confounding and different types of biases [19]. Addressing these challenges requires advanced analytical methods that account for real-world complexities and strengthen causal validity. Causal inference has evolved from its classical epidemiological foundation—rooted in confounding factors, biases and randomized trials [20, 21]—to an essential tool in modern machine learning (ML), where prediction alone is insufficient for decision-making [22]. Causal machine learning (CML), which integrates ML algorithms with causal inference principles to estimate treatment effects and counterfactual outcomes from complex, high-dimensional data [22, 23], plays a crucial role in overcoming RWD limitations by mitigating confounding and improving inference. CML has emerged as a promising approach for drug development. Unlike traditional ML, which excels at pattern recognition, CML aims to determine how interventions influence outcomes, distinguishing true cause-and-effect relationships from correlations, a critical factor for evidence-based decision-making [23, 24]. 

A key application of RWD/CML is estimating causal treatment effects in real-world settings by comparing treated and untreated patients. Propensity scores, traditionally estimated using logistic regression, have been widely used to mitigate bias through inverse probability weighting, matching, or covariate adjustment [25]. However, ML methods such as boosting, tree-based models, and neural networks regularly outperform logistic regression by better handling non-linearity and complex interactions [26–29]. More recently, deep representational learning has improved propensity score estimation in high-dimensional data [30, 31], while prognostic models enhance matching strategies, as described by Zhang et al. 2022. [32] Beyond propensity-based approaches, outcome regression models (e.g., G-computation) [33] directly adjust for confounding, leveraging RWD’s large sample sizes. Advanced techniques like targeted maximum likelihood estimation [34] and doubly robust inference [35] enhance causal estimation by combining outcome and propensity models, with ML improving predictive accuracy [36, 37]. Parallel to epidemiology, econometrics—where randomized experiments are often infeasible—leverages tools like instrumental variable analysis and structural equations [38], recently equipping them with deep learning and kernel-based methods [39–41]. Additionally, graphical modeling, as proposed by Pearl [42, 43], explicitly represents causal assumptions to refine treatment effect estimation [44, 45]. Pearl’s “do-calculus” aligns with potential outcomes in most cases but diverges in specific contexts, leading to ongoing academic debate [46]. Predictive modeling for clinical trial emulation has been validated by comparing its results to actual RCT outcomes, demonstrating its reliability [47]. 

This article introduces the transformative potential of applying CML to RWD in the context of drug development. Rather than focusing on a single methodological innovation, it provides a comprehensive overview of the key capabilities and outputs enabled by RWD/CML, emphasizing how these can complement and extend traditional clinical research by providing richer and more actionable insights into treatment effects. To ground this discussion, we present a series of use cases that illustrate the added value of integrating RWD/CML data with RCT data. While the promise of RWD/CML is considerable, it also brings technical, scientific, ethical, and regulatory challenges. The final section of the article critically evaluates these limitations and offers pragmatic recommendations aligned with current regulatory perspectives. By framing this integration within evolving scientific and regulatory paradigms, we aim to provide readers with a conceptual foundation for understanding the opportunities and challenges of this emerging field.

### Presentation of Selected Use Cases to Illustrate How CML on RWD Complement Traditional Clinical Trials Outputs and Enhance Clinical Development

In this section we illustrate how the integration of clinical trial data with RWD and CML can generate a more comprehensive and robust evidence base on drug effects. The series of cases illustrate how artificial intelligence can reveal clinically meaningful insights, for instance identifying patient subgroups or detecting delayed outcomes. These cases showcase a range of high-value outputs relevant to drug development, including trial emulation, increased efficiency in indication expansion, evaluation of treatment transportability, optimization of dosing strategies, and the design of innovative adaptive clinical trials. In certain contexts, RWD/CML can also facilitate the development of external control arms (ECAs), offering a rigorous alternative when traditional randomized controls are not feasible. Each use case has been chosen to highlight distinct contributions of RWD/CML across various domains—such as novel study designs, enhanced decision-making processes, or the generation of new evidence that supports multiple stakeholders. To emphasize the independent value of each contribution, the cases are presented as standalone examples without attempting to establish direct connections between them.

#### Identifying Subgroups and Refining Treatment Responses

A key advantage of RWD/CML is their ability to identify patient subgroups that demonstrate varying responses to a specific treatment. Predictors of treatment response may include for instance biomarkers, disease severity indicators, and longitudinal health status trends [48]. RWD offers a particularly valuable source of such information, often capturing a more comprehensive view of patient health than RCTs. By integrating these findings into predictive models, future trials can be better designed to target the most responsive patient populations, improving overall efficiency and effectiveness. This framework enhances RWD applications for clinical and regulatory decision-making and holds promise in advancing precision or personalized medicine by identifying patient subgroups that benefit most from specific treatments. Bertsimas et al. (2024) [49] introduced the R.O.A.D. framework, a method for clinical trial emulation using observational data while addressing confounding bias. Applied to 779 colorectal liver metastases patients, it accurately matched the JCOG0603 trial’s 5-year recurrence-free survival (35% vs. 34%). The approach uses prognostic matching and cost-sensitive counterfactual models to correct biases and identify subgroups with 95% concordance in treatment response. Traditionally, identification of patient subgroups requires testing for an interaction effect between treatment and patient attributes, which can reduce the power of the trial and may require multiple testing corrections [50]. ML models, in contrast, excel at scanning large datasets to detect complex interactions and patterns, making them particularly well-suited for discovering subpopulations with distinct responses [51–54]. In the RWD/CML approach it is also possible to deploy the outcome model’s predictions as a “digital biomarker,” effectively stratifying patients based on their predicted response and optimizing trial design accordingly [55]. 

#### Combining Information from RCT and RWD for a Comprehensive Drug Effect Assessment

Beyond patient stratification, RWD/CML enhances the integration of multiple data sources, maximizing the information derived from both RCTs and RWE. For instance, while RCTs provide robust short-term efficacy and safety data under controlled conditions, they often lack long-term follow-up, which can be supplemented by observational data from RWD sources. Such an approach is particularly useful for evaluating long-term treatment effects, identifying delayed adverse events, and assessing the sustainability of a drug’s benefits in real-life settings. Additionally, ECAs that incorporate both RCT and RWD data provide an alternative to traditional randomized comparisons. However, these approaches introduce unique biases, due to the absence of randomization and systematic differences between trial and real-world populations [56]. Bayesian power priors, which assign different weights to diverse evidence sources, offer a method for addressing these biases [57]. Other similar Bayesian methodologies have also been developed to integrate historical evidence into ongoing trials, even in cases where only aggregate data are available, such as in network meta-analyses [58], synthetic evidence approaches [58], or advanced Bayesian frameworks [59, 60]. 

#### Use of RWD/CML for Indication Expansion

Additionally, drugs approved for one condition often exhibit beneficial effects in other indications, and ML-assisted real-world analyses can provide early signals of such potential cross-indications [61, 62]. CML techniques (e.g., targeted maximum likelihood estimation, double machine learning, or Bayesian causal models) generate estimates of treatment effects while addressing confounding or selection biases. This can be achieved through spontaneous use tracking, assessment of incidental risk reduction in secondary conditions, or more systematic deep phenotyping approaches that integrate molecular knowledge graphs with RWD. For instance, Gao et al. (2022) [63] present KG-Predict, a knowledge graph-based computational framework for drug repurposing which enhances predictive accuracy drug discovery and repositioning.

#### RWD/CML for Estimating Transportability of Trial Results Across Populations

Another critical challenge in clinical research is the transportability of treatment effect estimates across different populations. Treatment effects identified in one cohort may not directly generalize to another due to differences in patient characteristics, disease prevalence, or healthcare settings. Adjusting for these “population shifts” is essential to ensure that findings from one study setting remain relevant elsewhere [64, 65]. In ML research, this issue is known as “concept drift” or “dataset shift,” and a rich body of evidence exists on methodologies to mitigate its impact [66]. Approaches such as *modified ensemble learning* have been specifically developed for clinical datasets, enhancing the robustness of predictive models when applied across diverse populations [67, 69]. One notable application of these techniques has been in dosing prediction, where ML-based models have been adapted to account for changing patient profiles and evolving clinical practices [68]. 

#### RWD/CML for Optimizing Dosage Determination

Finally, another crucial contribution of RWD/CML in advancing clinical trials consists in enhancing dosage optimization for improved efficacy and safety. Dosing decisions are traditionally based on phase 1 clinical data and preclinical models, which may lack the precision needed for real-world patient populations. By integrating real-world pharmacokinetic and pharmacodynamic data with predictive models, RWD-driven dose optimization strategies can improve the safety and efficacy of drug treatments. In certain therapeutic areas, natural variation in drug exposure post-approval can be analysed to refine dosing strategies and ensure optimal therapeutic effects while minimizing adverse events [69]. 

#### RWD/CML for Planning More Efficient Clinical Trials

The use of RWD is also central to describing natural history and prognostic research, which provides essential context for drug development. These studies offer insights into disease prevalence, severity, progression, and unmet medical needs. CML information is particularly valuable for refining trial eligibility criteria, identifying potential external controls, and guiding future modelling efforts. The ability to track real-world disease trajectories enables a deeper understanding of risk factors [70] and allows for the modelling of drug effects in complex clinical scenarios. Additionally, RWD/CML facilitates the study of competing risks, polypharmacy effects, and drug-drug interactions—critical considerations that are often underrepresented in RCTs, where patients typically have fewer comorbidities and concomitant medications [71]. 

#### RWD/CML for Designing Efficient Adaptive Trial and Sequential Learning

Adaptive clinical trial designs represent another major innovation facilitated by RWD/CML. Increasingly, clinical development programs incorporate adaptive strategies such as interim futility analyses [72] and dynamic enrolment adjustments [73]. Conceptually, the entire drug development process can be viewed as a sequential learning experiment, where each trial phase informs the next. RWD/CML plays a crucial role in this iterative process, allowing protocols to be refined in real-time based on accumulating data. In statistical and CML research, adaptive experiments have been formalized through frameworks such as multi-armed bandits [74] and Markov decision processes [75], which have gained widespread application in multiple domains, including clinical trials [76–78]. These approaches enable treatment allocation or enrolment criteria to be dynamically modified as new evidence emerges. In dose-finding phase 1 studies, for instance, ML-driven adaptive methodologies have been successfully applied to continuously reassess safety and efficacy, optimizing dosing regimens in real-time [79]. Hüyük et al. 2024 [80], demonstrated how ML-based optimization can refine trial decision-making, continuously evaluating whether a study should proceed, be adjusted, or be terminated early based on efficacy trends. Bayesian methodologies further support these dynamic decision processes, with growing regulatory acceptance of Bayesian designs in adaptive clinical trials [81–83], including applications in ECAs [84, 85]. In short, CML brings flexibility, precision, and learning capacity to adaptive trial design, turning RWD into a strategic resource for guiding and refining trials far beyond classical methods.

#### RWD/CML Generating External and Synthetic Control Arms

RWD/CML can play a key role in the generation of ECAs as valid comparators of single arm trials [86, 87]. The recent publication of guidelines by regulatory agencies on ECAs and synthetic control arms (SCAs) is an official recognition, which will likely facilitate their future use in clinical research, especially in oncology [88]. By leveraging data from real-world patient populations as a comparator group, ECAs or SCAs allow single-arm trials to generate comparative effectiveness estimates, particularly useful for rare diseases where randomization may be infeasible [18, 86, 89]. CML in this context plays a critical role to mitigate possible selection biases and control confounding factors [90, 91]. These controls can also be used internally for decision-making, enabling pharmaceutical companies to retrospectively enrich trial results with additional comparator data or to simulate potential future trial outcomes. The use of ECAs helps optimize go/no-go decisions, fine-tune upcoming trials, and refine statistical power calculations [86]. 

#### Using RWD/CML for Target Trial Emulation

Target trial emulation (TTE) is another emerging application of RWD/CML that transforms clinical trials, using a combined analysis of RWD and RCT data. TTE allows researchers to replicate or complement RCT results with RWD, while maintaining strict comparability in terms of inclusion/exclusion criteria and outcome definitions. The comparator arm can be simulated using real-world patients, whereas advanced modelling techniques can help model the treatment arm. This approach has proven valuable in improving alignment between RCT and real-world findings, reducing discrepancies caused by differences in study design [92]. At the same time, RWD can be leveraged to explore alternative hypotheses beyond the original scope of an RCT, therefore complementing traditional trial results. For instance to evaluate long-term outcomes, assess treatment effects in broader populations, or study rare subgroups where RCT evidence may be lacking [93]. The ability to emulate future trials also enhances clinical development by supporting interim decision-making, such as inclusion/exclusion refinements [94] and futility assessments [95]. 

### Challenges and Recommendations Regarding the Use of RWD/CML for Drug Development

The integration of AI into drug development is a groundbreaking advancement, with CML emerging as a powerful tool driving progress. The promise of RWD/CML quickly captured the attention of the pharmaceutical industry primarily due to their potential to address the inherent limitations of traditional clinical trials. However, the rapid rise of RWD/CML has been met with caution from regulatory agencies like the FDA and EMA. Additionally, the widespread adoption of RWD/CML faces additional technical and scientific challenges, such as the RWD quality and the complexity of algorithms requiring sophisticated programming. Furthermore, ethical concerns regarding data privacy, bias, and transparency continue to raise important questions about the responsible use of these technologies in drug development. The next section explores the technical, operational, and scientific challenges, followed by a discussion on the regulatory perspective of RWD/CML, as well as ethical and sociocultural considerations.

#### Technical/Operational and Scientific Challenges Faced by RWD/CML

CML applied to RWD faces several technical limitations that stem from both the nature of the data and the complexity of the models used. A major challenge is scalability and computational efficiency, as some CML methods require significant computational resources to process large, high-dimensional datasets typical of RWD. Organizations must invest in the infrastructure necessary to process and analyse RWD. One important concern regarding RWD/CML is the quality of the data used as RWD databases often suffer from missing data, inconsistent or incorrect coding, measurement errors, incomplete longitudinal follow-up, and biased patient selection. These limitations can undermine model validity and raise significant regulatory concerns. Currently, best practices emphasize a thorough documentation of source databases before their use, a resource-intensive process that requires transparent communication [96–98]. The development of standardized guidelines regarding technical equipment and operation is necessary to guide organizations that wish to enter this new discipline.

Beyond technical challenges, the scientific limitations of using RWD/CML stem from gaps in expertise and the absence of universally accepted best practices. The successful application of CML requires interdisciplinary knowledge that spans epidemiology, statistics, AI, and domain-specific expertise in healthcare or life sciences. Loftus (2024) [99] argues that progress in causal modeling has been hindered by scientific perfectionism and the lack of a human-centric approach. Hernán (2018) [100] emphasizes that causal analyses typically require not only good data and algorithms but also domain expert knowledge. Unlike traditional statistical methods that have well-defined guidelines for causal inference, CML is still evolving, and consensus on best practices for using these methods with RWD is still evolving. This gap in knowledge increases the risk of improper model selection, overfitting, and misattribution of causal effects. One important takeaway from this situation is the critical importance of rigorous validation of the CML generated models to ensure reliability, accuracy, and scientific integrity [101]. Baweja et al. 2023 [102] highlighted the challenges data scientists face in keeping up with the fast-paced advancements in ML, suggesting that human factors methods could be applied to address these difficulties. Addressing these scientific barriers requires comprehensive training programs, the development of standardized guidelines, and fostering collaboration across disciplines to build the necessary expertise for advancing CML applications in real-world settings.

#### Regulatory Perspective on Developing RWD/CML

The regulatory review of AI-generated results presents a significant factor shaping the path forward for this emerging technology. Both the FDA and EMA have been actively addressing the integration of AI and RWD in drug development by progressively developing frameworks and guidelines to balance innovation with regulatory rigor. The FDA first acknowledged the utility of RWE/RWD to complement clinical trials in 2017, particularly for complementing clinical trials in specialized contexts such as oncology [98]. However, the use of CML on RWD introduces regulatory concerns, particularly regarding the transparency of AI-driven models. Many CML models function as “black boxes,” offering little insight into their internal decision-making processes. This opacity raises concerns about the reproducibility of scientific findings and the trustworthiness of AI-generated evidence, especially in regulatory decision-making, where understanding the rationale behind conclusions is crucial [103]. Recognizing these challenges, the FDA released its first set of recommendations on AI in drug development in January 2025. This guidance introduced a risk-based framework for sponsors to assess and establish the credibility of AI models within defined contexts of use, outlining necessary steps to ensure model reliability and applicability [104]. In Europe, the EMA published a reflection paper on AI in drug development in September 2024, addressing key issues such as data quality, model validation, and the ethical implications of AI deployment in clinical research. This document underscores the agency’s commitment to ensuring that AI applications are both scientifically sound and ethically justified [105]. These regulatory efforts reflect a broader commitment to balancing patient safety, product efficacy, and innovation in the rapidly evolving field of AI-driven drug development. They also underscore the responsibility on the research teams to develop high-quality models that are transparent and can be reproducible—an effort that, in practice, requires addressing the challenges outlined in section “*Technical/Operational and Scientific Challenges”.*

#### Ethical Challenges in RWD/CML Integration

The integration of RWD and CML into drug development also raises significant ethical concerns, particularly regarding privacy, consent, and data ownership [106, 107]. Since RWD is often derived from EHRs, insurance claims, and other sensitive sources, compliance with privacy regulations, such as the Health Insurance Portability and Accountability Act (HIPAA) in the U.S. and the General Data Protection Regulation (GDPR) in Europe, is essential to protect patient confidentiality. However, the use of large-scale datasets required for AI and CML modelling, along with extensive exploratory analyses of the variable relationships through causal networks, increases the risk of re-identifying anonymized patient records, potentially leading to privacy breaches [108]. Additionally, ambiguities surrounding data ownership—whether it belongs to patients, healthcare institutions, or third-party entities—can lead to disputes over access, usage rights, and potential commercialization, further complicating the ethical landscape [108]. Establishing robust ethical frameworks is crucial to ensuring that AI-driven healthcare solutions uphold ethical principles while enhancing patient care [109]. 

A final critical barrier to AI adoption in drug development is sociocultural, driven by skepticism among healthcare professionals and patients toward AI-generated recommendations. Concerns over the fairness, transparency, and potential biases of algorithmic decisions can foster public reluctance and mistrust, slowing widespread acceptance. Addressing these challenges requires clear communication, robust accountability measures, and proactive efforts to ensure diverse representation in research. Moreover, since RWD encompasses individuals from diverse cultural, socioeconomic, and healthcare backgrounds, CML models must be designed to account for these differences, ensuring that treatment recommendations are both equitable and culturally relevant. Building trust in AI-driven applications depends on ongoing collaboration with healthcare providers, active patient engagement, and a steadfast commitment to transparency.

## Conclusions

This paper has highlighted the rapid development of CML and RWD as complementary tools in drug development, addressing limitations of traditional clinical trials. CML enables unbiased drug effect estimation, responder subgroup identification, and long-term efficacy and safety assessment. It also supports clinical trial adaptation and external control generation. However, widespread adoption is hindered by the limited quality of some source-databases, scarce relevant technical expertise, lack of standardized guidelines, and universally accepted best practices. Overcoming these barriers requires multidisciplinary collaboration to establish rigorous methodologies, develop operational guidelines, and integrate these approaches into widely used software packages. Regulatory agencies are progressively providing guidance and structured initiatives to evaluate these technologies responsibly. Achieving broader acceptance and trust, however, requires transparent communication with stakeholders regarding how these models are developed, tested, and applied. These methods should only be implemented by institutions with the necessary infrastructure and expertise to ensure methodological rigor, transparency, and reproducibility. By fostering stakeholder engagement and establishing rigorous, evidence-based implementation strategies, these innovative approaches can complement RCTs and accelerate the future of drug development programs and regulatory science.

## Data Availability

No datasets were generated or analysed during the current study.
